# Perceived Teacher Autonomy Support and School Engagement of Tibetan Students in Elementary and Middle Schools: Mediating Effect of Self-Efficacy and Academic Emotions

**DOI:** 10.3389/fpsyg.2020.00050

**Published:** 2020-01-31

**Authors:** Wei Li, Wenyang Gao, Jingrong Sha

**Affiliations:** ^1^School of Education Science and Technology, Northwest Minzu University, Lanzhou, China; ^2^School of Management, University of Science and Technology of China, Hefei, China

**Keywords:** Tibetan areas, elementary and middle school students, teacher autonomy support, self-efficacy, academic emotions, school engagement

## Abstract

School engagement (SE) refers to the intensity and quality of emotions experienced by students when commencing and carrying out learning activities, and includes behavioral, emotional, and cognitive engagement. A high SE level promotes academic achievement, reduces students’ behavioral problems, and prevents school dropout. This study, whose participants were 819 students from Tibetan areas, explored the impact of teacher autonomy support (TAS) on students’ SE and the mechanisms involved in this relationship. The results showed that TAS had a positive impact on SE, while students’ self-efficacy had a mediating effect between TAS and SE. On the one hand, TAS affected self-efficacy through academic interest and ultimately influenced SE; moreover, TAS negatively affected academic anxiety, indirectly inhibiting the negative effect of academic anxiety on SE through self-efficacy. The theoretical and practical implications of the study findings are discussed.

## Introduction

School engagement (SE) refers to the intensity and quality of emotions experienced by students when commencing and carrying out learning activities (Fredricks et al., 2004; Wen et al., 2010). Studies on the structure of SE have proposed different structures and dimensions. Initially, Schaufeli and Bakker (2004) considered that SE included vigor, dedication, and absorption. On this basis, increasingly more studies defined SE as comprising behavioral, emotional, and cognitive engagement (Jimerson et al., 2003; Fredricks et al., 2004; Appleton et al., 2008). Behavioral engagement corresponds to students’ involvement in academic and social aspects of school; the emotional component includes students’ affective reactions toward their school experiences, and whether they enjoy or dislike academic activities; cognitive engagement refers to the use of metacognitive and cognitive strategies, and also includes students’ willingness to try hard and put effort into understanding complex academic tasks (Archambault et al., 2017). SE including these three aspects can better explain the SE of students. For example, some students have a high behavioral engagement but their academic performance is not good.

School engagement has been found to promote academic achievement (Klem and Connell, 2004; Upadyaya and Salmela-Aro, 2013). Conversely, students with low SE levels are more likely to experience negative emotions such as depression (Upadyaya and Salmela-Aro, 2013; Vaughn et al., 2013), display more negative behaviors such as absenteeism (Rumberger and Lim, 2008; Henry et al., 2012), and even drop out of school (Fall and Roberts, 2012; Rumberger and Rotermund, 2012; Liu et al., 2018). Therefore, SE is considered an effective indicator of dropout risk (Dryfoos, 1990).

Many studies in China have explored the mechanism behind the SE of middle school students (Chen et al., 2015; Li, 2018; Wang et al., 2019), as well as the status of SE among middle and high school students in Northwest China (An et al., 2007; Maslak et al., 2010). However, there are few studies on the SE of elementary and middle school students in China’s Tibetan areas. One such study was conducted by Li et al. (2017), which involved 6,000 families living in rural Western China, and found that children in religious families were more likely to drop out of school than those in non-religious families. This was especially the case for children from families that practiced Tibetan Buddhism. Considering that SE is a powerful predictor of school dropout, research on the SE of Tibetan elementary and middle school students would be helpful for estimating their potential dropout risk. Furthermore, understanding the factors affecting Tibetan students’ SE can also provide ideas and suggestions for increasing SE in this population.

### Teacher Autonomy Support and SE

Researchers have used a variety of motivational theories and models to explain the factors affecting SE, including the theories of self-determination, self-regulation, goals, and expected value (Fredricks et al., 2016). Among these, the self-determination theory has been widely applied (Reeve, 2009, 2012). Based on this theory, teacher autonomy support (TAS) refers to an individual’s perception that his/her views are supported and recognized by the teacher, who also provides him/her with opportunities to access information and make choices (Mageau and Vallerand, 2003). TAS is an important socioenvironmental factor emphasized in the self-determination theory (Chen et al., 2015). The student-teacher framework in this theory clearly points out that the relationship between teachers and students has the dual role of cultivating or obstructing students’ SE and learning motivation. Specifically, a good teacher-student relationship is conducive to the promotion of students’ SE (Fredricks et al., 2016). A large number of studies have confirmed the positive impact of TAS on students’ SE (Reeve et al., 2004; Jang et al., 2010; Chen et al., 2015; Hospel and Galand, 2016; Sun, 2016; Zhang, 2018; Martin and Collie, 2019).

Students shape their self-awareness, emotions, and behaviors by perceiving and interpreting information in their social environments (Vedder et al., 2005), and TAS plays a vital role in the formation of a positive and interactive relationship between students and the school (Eccles et al., 1993; Deci, 2009). Since interpersonal relationships are seen as the basis for motivational development, students will internalize and accept the values of those individuals who provide them with positive emotional support and with whom they are closely connected (Ryan and Deci, 2000; Kenny and Bledsoe, 2005).

When teachers demonstrate supportive behaviors toward their students, such as supporting their autonomy, showing high expectations of them, giving them continuous and clear feedback, and providing them with varied, challenging, interesting, and meaningful tasks (Fredricks et al., 2016), students will be encouraged to develop good learning habits (Slater et al., 2012), which in turn will generate adequate learning behaviors (Chai et al., 2011; Cooper, 2014). On the contrary, student-teacher conflict leads to lower behavioral engagement (Engels et al., 2019). Based on the above, this study proposed the following hypothesis.

H1: Perceived TAS is positively associated with the SE of Tibetan students.

### Mediating Effects of Self-Efficacy

The theory of self-determination proposed by Ryan and Deci (2000) assumes that the key to students’ learning motivation lies in the satisfaction of three basic psychological needs, namely the needs for competency, affiliation, and autonomy. Teachers’ supportive behaviors fulfill students’ needs for competency and affiliation (Jin and Wang, 2019). Examples include actively understanding students’ learning situations and ideas; giving them sufficient freedom and support in the arrangement of academic tasks, selection of learning contents, and the methods for solving problems; and minimizing the use of coercive and demanding methods when teaching (Deci and Ryan, 1985; Stefanou et al., 2004; Lam et al., 2009). When students’ psychological needs are satisfied, this is conducive to their development of self-efficacy, defined as the individual’s judgment and evaluation of the degree of completion before completing a specific task, and the degree of mastery of their ability to achieve their goals (Bandura, 1977). For example, when teachers discuss solutions to problems with students and respect the ideas that they share, their need for competency is satisfied; this promotes the internalization of their motivation and enhances their autonomy to participate in learning (Zhang et al., 2018), which in turn improves their self-efficacy (Hardre and Reeve, 2003) and SE level (Jang et al., 2010).

There is a significant and positive correlation between self-efficacy and academic behaviors (Alivernini and Lucidi, 2011; Yu and Singh, 2018). Previous studies have also confirmed that students’ self-efficacy plays a mediating role between teachers’ behaviors and their learning performance. In a study by Wang et al. (2017) with 637 Chinese middle school students, self-efficacy had a mediating effect between TAS and learning engagement in math. Considering the above findings, this study proposed the following hypothesis.

H2: Self-efficacy plays a mediating role between the perceived TAS and SE of Tibetan students.

### Mediating Effect of Academic Emotions

Pekrun et al. (2007) defined academic emotions as those experienced by students in relation to their learning activities and results. As a non-intellectual factor, academic emotions affect students’ learning satisfaction, attention, learning strategies, and cognitive resources (Yu and Dong, 2007; Lewis et al., 2009). At the same time, positive academic emotions can improve cognitive flexibility, self-regulation ability (Eynde et al., 2006), and learning engagement (Lewis et al., 2009). TAS is conducive to the cultivation and stimulation of academic interest (Yu and Singh, 2018). Students tend to show a stronger interest in learning when they perceive that their teachers are supportive resources (Chen et al., 2015). There will also be a greater sense of self-efficacy and a higher expectation of successful learning behaviors (Sakiz et al., 2012; Yu and Singh, 2018). Reversely, TAS plays an important role in inhibiting students’ negative emotions. A longitudinal study of middle school students found that TAS was significantly and negatively correlated with their level of anxiety and depression 1 year later (Yu et al., 2016). Other related studies have also verified the negative relationship between perceived TAS and students’ feelings of anxiety and depression (Way et al., 2007; LaRusso et al., 2008; Federici and Skaalvik, 2014; Nilsen, 2018).

Students’ negative emotions have a dampening effect on SE. Assor et al. (2005) conducted a study with 319 elementary school students in grades 45 and found that angry and anxious feelings increased the motivation defined as the lack of intention or volition to put in any effort (i.e., A-motivation) and the motivation only concerned with punishments and rewards (i.e., extrinsic motivation), which curbed SE. Therefore, TAS may suppress the negative effect that academic anxiety has on SE. Based on this, the following hypothesis was proposed.

H3: Perceived TAS affects the SE of Tibetan students through academic emotions. On the one hand, TAS positively influences academic interest to promote SE; on the other hand, TAS negatively influences academic anxiety, in turn curbing the negative impact of academic anxiety on SE.

## Materials and Methods

### Participants

The participants of this study were 1,000 middle school students from two cities in the Gannan Tibetan Autonomous Prefecture of Gansu Province. Students were selected using convenience sampling. After the questionnaire was collected, a total of 181 questionnaires were excluded, as they included a large number of unanswered questions or showed a clear tendency to respond in a regular manner (e.g., extreme response tendency). Thus, there were 819 valid questionnaires, representing an effective recovery rate of 81.9%. The number of participants from the two cities was 452 and 367, respectively. A total of 658 participants came from families with more than one child, 100 were from one-child families, and 61 participants did not answer this question. Participants’ average age was 14.63 years (*SD* = 1.22), with the age range being 11–18 years. There were 477 males (58.2%), 318 females (38.8%), and 24 (2.9%) who did not specify their gender. Moreover, 173 (21.1%) were in grade 5, 324 (39.6%) in grade 6, 286 (34.9%) in junior middle school, and 36 (4.4%) did not report their grade. The majority were Tibetans, totaling 796 (97.2%), 2 (0.2%) had another ethnicity, and 21 (2.6%) did not report their ethnicity. We conducted a one-way analysis of variance (ANOVA) to compare mean differences between the grades for all the variables. The results demonstrated that students differed significantly in academic anxiety, *F*(2,780) = 9.658, *p* < 0.001, $\eta_{\text{p}}^{2}$ = 0.024, such that students in junior middle school scored higher on academic anxiety than did students in elementary school. The results also showed that students differed significantly in terms of SE, *F*(2,780) = 4.942, *p* = 0.007, $\eta_{\text{p}}^{2}$ = 0.013, with students in grade 6 scoring higher on SE than did students in grade 5 and junior middle school. There were no significant differences between the grades in terms of TAS, *F*(2,780) = 0.233, *p* = 0.792; academic interest, *F*(2,780) = 1.773, *p* = 0.171; or self-efficacy, *F*(2,780) = 0.505, *p* = 0.604.

### Procedure

Approval was obtained from the Northwest Minzu University’s academic ethics committee prior to the commencement of the study. Before the survey was conducted, the students were informed of the anonymous nature of the data collection and analysis. Informed consent forms were also distributed to the students and their parents. Trained researchers provided instructions before the students filled in the questionnaire, after which students recorded their demographic information and answered the questionnaire items. All completed questionnaires were collected on the spot.

### Measures

#### Teacher Autonomy Support

This study used the autonomy support scale developed by Shi (2009), which was revised from the original scale compiled by Williams and Deci (1996). The scale measured students’ perceived level of TAS during class and consisted of six items (e.g., “Most teachers believe that I have the ability to properly learn the material taught in school”). Scoring was based on a 6-point Likert scale, with 1 and 6 representing “fully disagree” and “fully agree,” respectively. The model fitting results of confirmatory factor analysis were good: χ^2^ = 57.439, degrees of freedom (*df*) = 9, χ^2^/*df* = 6.382, comparative fit index (CFI) = 0.961, Tucker-Lewis index (TLI) = 0.935, root mean square error of approximation (RMSEA) = 0.081, standardized root mean squared residual (SRMR) = 0.030. The composite reliability (CR) and average variance extracted (AVE) of the TAS measure were 0.796 and 0.397, respectively. Cronbach’s α for the TAS scale in this study was 0.80.

#### Academic Interest and Anxiety

This study focused on two academic emotions only: academic interest and academic anxiety. The corresponding dimensions in the academic emotions scale compiled by Lai (2013) were used. The items focused on learning and classroom experiences (e.g., “Learning math makes me feel happy” for academic interest, and “I feel anxious about learning and doing well in math” for academic anxiety). There were six items for each dimension, scored on a 6-point Likert scale (1 = “fully disagree”; 6 = “fully agree”). For each participant, the average of the scores on all items in a subscale was the score for that subscale. The model fitting results of confirmatory factor analysis were good for both academic interest (χ^2^ = 113.020, *df* = 9, χ^2^/*df* = 12.558, CFI = 0.963, TLI = 0.939, RMSEA = 0.119, SRMR = 0.028) and academic anxiety (χ^2^ = 123.175, *df* = 9, χ^2^/*df* = 13.686, CFI = 0.930, TLI = 0.883, RMSEA = 0.124, SRMR = 0.045). The CR and AVE were 0.901 and 0.605, respectively, for academic interest, and 0.830 and 0.451, respectively, for academic anxiety. In this study, Cronbach’s α for the dimensions of academic interest and anxiety was 0.90 and 0.83, respectively.

#### Self-Efficacy

The self-efficacy scale used in this study was compiled by Wu and Cheng (1992) and contained five items (e.g., “I am certain that I can understand the most difficult section in the math textbook”), scored on a 6-point Likert scale (1 = “fully disagree”; 6 = “fully agree”). The model fitting results of confirmatory factor analysis were good (χ^2^ = 42.559, *df* = 5, χ^2^/*df* = 8.512, CFI = 0.970, TLI = 0.940, RMSEA = 0.096, SRMR = 0.027). The CR and AVE for self-efficacy were 0.815 and 0.470, respectively. In the current study, Cronbach’s α for the self-efficacy scale was 0.81.

#### School Engagement

The SE questionnaire used in this study was compiled by Awang et al. (2008) and contained a total of 29 questions, encompassing the three dimensions of behavioral, cognitive, and psychological participation. Example items for the respective dimensions are “I forgot to bring my exercise book and stationery to class,” “I will prepare some questions in advance so that I can concentrate on learning the lesson,” and “I feel that I am a member of the school.” Items were scored on a 5-point Likert scale (1 = “fully disagree”; 5 = “fully agree”). The model fit results of confirmatory factor analysis were not good (χ^2^ = 1351.877, *df* = 374, χ^2^/*df* = 3.615, CFI = 0.799, TLI = 0.782, RMSEA = 0.057, SRMR = 0.063). The CR for behavioral, cognitive, and psychological participation was 0.821, 0.786, and 0.680, respectively. The AVE for behavioral, cognitive, and psychological participation were 0.368, 0.273, and 0.177, respectively. The composite reliability for the overall SE was 0.836, 95% confidence interval (CI) [0.813, 0.859]. Cronbach’s α for behavioral, cognitive, and psychological participation was 0.82, 0.78, and 0.69, respectively; and Cronbach’s α for the overall SE scale was 0.80.

### Data Analysis

First, the Expectation-Maximization (EM) algorithm was used to fill in the missing values. Next, Mplus7.4 was used to test the various research hypotheses, with the estimation method used being the maximum likelihood estimation (Muthén and Muthén, 1998–2015). Based on recommendations from previous research (Bandalos, 2008; Wu and Wen, 2011), the questions in the SE scale were packaged using the internal consistency method, meaning that questions under the same dimension were packaged (Little et al., 2002). The model fit was jointly measured using five indicators: χ^2^/*df*, CFI, TLI, RMSEA, and SRMR. The following values indicated a good fit: χ^2^/*df* < 3, CFI > 0.90, TLI > 0.90, RMSEA < 0.08, and SRMR < 0.08 (Marsh et al., 2004). In addition, 2,000 bootstrap samples were constructed to calculate the point estimate of the mediating effect and the 95% CI (Preacher and Hayes, 2008). Raw data file for this article could be found in the Supplementary Material.

## Results

### Test for Common Method Variance

Harman’s single-factor method was used to test for CMV (Podsakoff et al., 2003), and unrotated exploratory factor analysis was performed on all the questions. The results showed that 12 factors had an initial eigenvalue greater than 1. The variance explained by the first factor was 15.285%. This was much lower than the critical value of 40%, indicating that the CMV was not obvious in this study.

### Descriptive Statistics and Correlation Between Variables

Values of skewness and kurtosis, means, standard deviations, and correlation coefficients are presented in Table 1. All variables displayed acceptable ranges (i.e., Skewness and Kurtosis < | 0.35|) of skewness and kurtosis (Trochim and Donnelly, 2006; George, 2011). The correlations were significant and positive for the following relationships: SE with TAS, self-efficacy with academic interest, TAS with self-efficacy and academic interest, and self-efficacy with academic interest. The correlations for academic anxiety with TAS, self-efficacy, academic interest, and SE were significant and negative to varying degrees.

**TABLE 1:** Descriptive statistics and correlation matrix of research variables.

Variables	1	2	3	4	5
(l) TAS	1				
(2) Self-efficacy	0.41***	1			
(3) Academic interest	0.44***	0.43***	1		
(4) Academic anxiety	−0.13***	−0.23**	−0.33***	1	
(5) SE	0.21***	0.25***	0.25***	−0.22***	1
Mean	3.70	3.58	3.67	3.23	3.44
Standard deviation	1.00	1.00	1.16	1.08	0.46
Skewness	–0.21	–0.08	–0.17	0.07	0.22
Kurtosis	–0.21	–0.17	–0.34	–0.53	–0.20

**p < *0.01*, ***p < *0.001*; 1 = Teacher autonomy support (TAS); 2 = Self-efficacy; 3 = Academic mterest; 4 = Academic anxiety; and 5 = School engagement (SE).

### Results of Multiple Mediation Model

**FIGURE 1:**
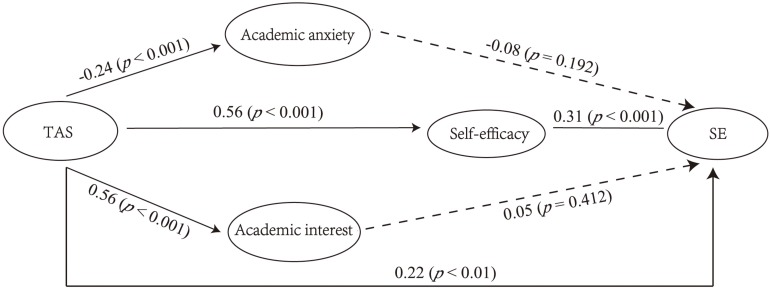
Results of model with academic anxiety, academic interest, and self-efficacy as mediators (Model 1) including standardized coefficients (TAS = teacher autonomy support; SE = school engagement).

First, the mediating effects of academic anxiety, self-efficacy, and academic interest were examined, and the results are shown in Figure 1 (Model 1). The model fitting results were good (χ^2^ = 1000.954, *df* = 292, χ^2^/*df* = 3.428, CFI = 0.911, TLI = 0.901, RMSEA = 0.054, and SRMR = 0.068). As can be seen from the figure, TAS had a statistically significant effect on academic anxiety (*B* = −0.41, *SE* = 0.10, *p* < 0.001; β = −0.24, *SE* = 0.05, *p* < 0.001), self-efficacy (*B* = 0.77, *SE* = 0.10, *p* < 0.001; β = 0.56, *SE* = 0.04, *p* < 0.001), and academic interest (*B* = 1.05, *SE* = 0.12, *p* < 0.001; β = 0.56, *SE* = 0.04, *p* < 0.001). Academic anxiety and academic interest did not have a significant effect on SE (*B* = −0.01, *SE* = 0.01, *p* = 0.441; β = −0.08, *SE* = 0.06, *p* = 0.192; and *B* = 0.004, *SE* = 0.01, *p* = 0.569; β = 0.05, *SE* = 0.06, *p* = 0.412, respectively). Self-efficacy had a significant impact on SE (*B* = 0.04, *SE* = 0.02, *p* < 0.05; β = 0.31, *SE* = 0.07, *p* < 0.001). Only self-efficacy played a mediating role between TAS and SE: the estimated value of the mediating effect ab was 0.029 (*SE* = 0.014, *p* < 0.05, 95% CI [0.01, 0.06]). The standardized estimated value of the mediating effect ab was 0.174 (*SE* = 0.042, *p* < 0.05, 95% CI [0.10, 0.26]).

**FIGURE 2:**
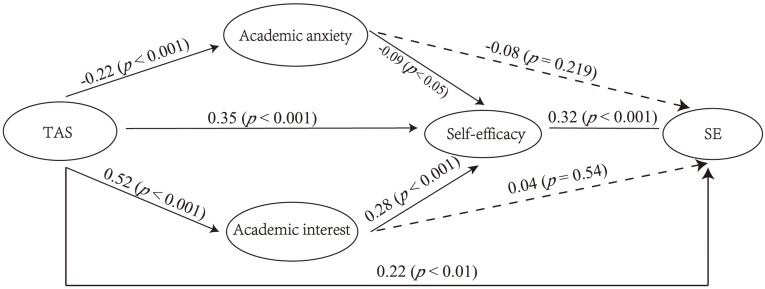
Results of model with academic anxiety, academic interest, and self-efficacy as mediators (Model 2) including standardized coefficients (TAS = teacher autonomy support; SE = school engagement).

Next, we investigated whether TAS affected self-efficacy through academic anxiety and academic interest, thereby further affecting SE. The results are shown in Figure 2 (Model 2). The model fitting results were good (χ^2^ = 949.014, *df* = 290, χ^2^/*df* = 3.272, CFI = 0.917, TLI = 0.907, RMSEA = 0.053, and SRMR = 0.061). As can be seen from Figure 2, TAS significantly affected academic anxiety (*B* = −0.37, *SE* = 0.09, *p* < 0.001; β = −0.22, *SE* = 0.05, *p* < 0.001), self-efficacy (*B* = 0.48, *SE* = 0.09, *p* < 0.001; β = 0.35, *SE* = 0.05, *p* < 0.001), and academic interest (*B* = 0.98, *SE* = 0.11, *p* < 0.001; β = 0.52, *SE* = 0.04, *p* < 0.001). Self-efficacy was separately affected by academic anxiety (*B* = −0.07, *SE* = 0.03, *p* < 0.05; β = −0.09, *SE* = 0.04, *p* < 0.05) and academic interest (*B* = 0.21, *SE* = 0.04, *p* < 0.001; β = 0.28, *SE* = 0.06, *p* < 0.001). In turn, self-efficacy had a significant impact on SE (*B* = 0.04, *SE* = 0.02, *p* < 0.05; β = 0.32, *SE* = 0.07, *p* < 0.001). Academic anxiety and academic interest had no significant effect on SE (*B* = −0.008, *SE* = 0.01, *p* = 0.461, β = −0.08, *SE* = 0.06, *p* = 0.219; and *B* = 0.003, *SE* = 0.01, *p* = 0.662, β = 0.04, *SE* = 0.06, *p* = 0.54, respectively). Thus, TAS had a significant impact on SE through self-efficacy directly, or indirectly through academic anxiety or academic interest affecting self-efficacy.

We compared Models 1 and 2 using chi-square. The result was significant (Δχ^2^ = 51.94, Δ*df* = 2, *p* < 0.001) which can be seen from Table 2; thus, it is necessary to consider the effect of both academic anxiety and academic interest on self-efficacy.

**TABLE 2:** Comparison of models 1 and 2.

Model	*χ*^2^	*df*	*χ*^2^*/df*	CFI	TLI	RMSEA	SRMR	Δ*χ*^2^	Δ*χdf*
Model 1	1000.954	292	3.428	0.911	0.901	0.045	0.068	51.94	2
Model 2	949.014	290	3.272	0.917	0.907	0.053	0.061		

**df* = degrees of freedom; CFI = comparative fit index; TLI = Tucker-Lewis index; RMSEA = root mean square error of approximation; SRMR = standardized root mean squared residual.*

## Discussion and Conclusion

### Direct Effect of TAS on SE

This study investigated the ways by which TAS, academic emotions, and self-efficacy affected the SE of Tibetan elementary and middle school students. The results showed that TAS could directly affect students’ SE, which was consistent with the study hypothesis H1 and previous research findings (Sakiz et al., 2012; Yu and Singh, 2018). The ecosystem theory (Bronfenbrenner, 2009) postulates that a school is a microsystem with a direct impact on students’ development; teachers play a vital role in this microsystem, and their frequent interactions with students can subtly influence the latter’s academic performance and behaviors.

Individual’s motivating styles could have impact on one’s motivations, emotions, and learning performance (Deci and Ryan, 1985), and teachers with high TAS levels often engage in the following motivating styles: (i) fostering internal motivational resources; (ii) relying on information-rich and non-controlling language; (iii) communicating the importance and justifications when scheduling tasks; and (iv) recognizing and accepting students’ expressions of negative emotions and adopting supportive behaviors such as carefully listening to them (Reeve, 2006). These TAS behaviors not only satisfy students’ psychological needs, but also stimulate their internal learning motivations (Reeve et al., 2004). Thus, when they participate in learning activities, they become more concerned about the activities’ inherent interest; this makes them more willing to invest time and effort into the learning tasks, which increases their SE levels.

### Indirect Effect of TAS on SE Through Self-Efficacy

The study found that self-efficacy played a mediating role between TAS and SE, while academic interest and academic anxiety did not; thus, hypothesis H2 was proven and hypothesis H3 was disproven. When teachers respect students and their ways of thinking and allow them to express their views when they encounter problems, students perceive TAS and respect. This strengthens the affirmation of their own abilities and enhances their self-efficacy (Bandura, 1997), which would not become negatively affected when they encounter problems that cannot be solved or when their solutions are erroneous (Yu and Singh, 2018). Thus, a strong sense of self-efficacy directly stimulated students’ academic interest and increased their SE levels.

The lack of teachers in Tibetan areas has long been a problem. In recent years, the state has intensified the training of teachers in minority areas and diversified the recruitment channels, such that actual teaching needs have basically been met in terms of the total number of teachers needed. However, there is still a severe shortage of teachers who are proficient in bilingual teaching skills to fill the positions in bilingual schools for ethnic minorities. In this scenario, existing teachers focus more of their energy on preparing and teaching lessons, and completing the various basic teaching tasks. In the process, they are often unable to concurrently consider students’ feelings and needs.

When teachers respect students, show them concern, and allow them to express their views, such efforts will be conducive to enhancing their self-efficacy. This in turn makes them more confident about their abilities to solve and complete learning tasks independently, thereby further enhancing their enthusiasm and initiative in SE. On the contrary, when TAS is lacking, students are more likely to regard their difficulties in learning as threats. The manifestation of such a behavior is precisely due to the lack of self-efficacy. The result will be a corresponding lack in SE (Jiang et al., 2019).

### Indirect Effect of TAS on SE Through Academic Emotions and Self-Efficacy

In addition, the study found that TAS did not simply affect SE through academic interest, but also further enhanced SE by boosting students’ self-efficacy through academic interest. TAS improved students’ internal motivation, which naturally triggered their academic interest. SE cannot be enhanced by the students having pure academic interest or simply being passionate about learning. With regard to learning, the common sayings are to “set long-term goals rather than frequent ones” and “avoid doing things by fits and starts.” Individuals’ SE levels are really elevated only when they have a positive interest in learning and are able to positively estimate the successful achievement of their intended goals. TAS can satisfy the students’ basic psychological needs. When that happens, students perceive teachers’ concern for them, which in turn heightens their level of positive emotions (Li, 2018). The rise in positive emotions makes students more enthusiastic, confident, and focused on completing their academic activities; thus, their SE is further elevated.

This study also found that TAS reduced students’ academic anxiety. Previous studies have similarly identified that an active teacher-student relationship reduced the symptoms of anxiety and depression in adolescents (Krane et al., 2017). The fulfillment of the three basic psychological needs proposed by the self-determination theory help to maintain a person’s mental health (Ryan and Deci, 2000). TAS plays a critical role in satisfying those three needs in students, thus effectively reducing their negative emotions. This study further found that students’ academic anxiety did not directly affect their SE. Rather, it was an indirect effect through self-efficacy. This might be because appropriate levels of anxiety during learning could sustain the excitement of learning, thereby strengthening learning initiative and consciousness (Wang and Cheng, 2005). However, excessive levels of anxiety would be counterproductive. TAS reduces students’ academic anxiety, and low levels of anxiety help them make subjective positive estimates regarding the results of their learning activities. This improves their self-efficacy in learning, thus increasing their SE.

Academic interest and anxiety did not affect SE directly, but through self-efficacy. This might be because emotions are important media for expressing motivational information (Assor et al., 2005), meaning that varying emotions might trigger different motives. Assor et al. (2005) found that teachers’ controlling behaviors generated negative emotions in students; these triggered A-motivation and extrinsic motivation, which further inhibited SE.

Self-efficacy is an important factor for stimulating motivation, and students with high self-efficacy have stronger academic motivations. Students with greater academic interest believe that they are able to complete their academic tasks well, and are more likely to demonstrate stronger academic motivations, thus stimulating their SE levels. In contrast, students with high levels of academic anxiety are less confident about completing their academic tasks. They have lower academic motivation, which is not conducive to the generation of SE. Therefore, in addition to increasing students’ academic interest during teaching activities, teachers should also reduce students’ academic anxiety and promote their sense of self-efficacy.

### Limitations and Future Research

This study has several shortcomings that can be addressed in future research. First, convenient sampling was used to select Tibetan students from two cities in Gannan Prefecture, Gansu Province; thus, the sample may lack representativeness. In future research, the sample can be larger and more representative by selecting Tibetan students from the entire northwestern region and other Tibetan autonomous regions. Second, this study used a cross-sectional research design, which limits the possibility to make causal inferences. Moreover, the variables being measured were self-reported by students and not by their teachers, which might have caused common method bias. A longitudinal study design and multi-source evaluations should thus be adopted in future research. Last, this study did not take into consideration the students’ class factors, their respective schools, and other high-level factors. School education is an organic system, and factors at different levels have important effects on students’ attitudes and behaviors; therefore, such factors should be fully considered in future research.

## Data Availability Statement

All datasets generated for this study are included in the article/Supplementary Material.

## Ethics Statement

The studies involving human participants were reviewed and approved by the Research Ethics Committee, School of Education Science and Technology, Northwest Minzu University. Written informed consent to participate in this study was provided by the participants’ legal guardian/next of kin.

## Author Contributions

WL contributed to data analysis and writing of the whole manuscript. WG contributed to data analysis and editing of the manuscript. JS contributed to study design and data collection.

## Conflict of Interest

The authors declare that the research was conducted in the absence of any commercial or financial relationships that could be construed as a potential conflict of interest.
